# Knowledge, attitude and practice of healthcare workers on infection prevention and control in Ethiopia: A systematic review and meta-analysis

**DOI:** 10.1371/journal.pone.0308348

**Published:** 2024-09-05

**Authors:** Chalachew Adugna Wubneh, Birye Dessalegn Mekonnen, Tewodros Getaneh Alemu, Masresha Asmare Techane, Nega Tezera Assimamaw, Getaneh Mulualem Belay, Tadesse Tarik Tamir, Addis Bilal Muhye, Destaye Guadie Kassie, Amare Wondim, Bewuketu Terefe, Bethelihem Tigabu Tarekegn, Mohammed Seid Ali, Beletech Fentie, Almaz Tefera Gonete, Berhan Tekeba, Selam Fisiha Kassa, Bogale Kassahun Desta, Amare Demsie Ayele, Melkamu Tilahun Dessie, Kendalem Asmare Atalell

**Affiliations:** 1 Department of Pediatrics and Child Health Nursing, School of Nursing, College of Medicine and Health Science, University of Gondar, Gondar, Ethiopia; 2 Department of Nursing, Teda health Science College, Gondar, Ethiopia; 3 Community Health Nursing Unit, School of Nursing, College of Medicine and Health Science, University of Gondar, Gondar, Ethiopia; University of Brasilia: Universidade de Brasilia, BRAZIL

## Abstract

**Introduction:**

Infectious diseases remain the leading causes of death in low and middle-income countries including Ethiopia. The existence of emerging, re-emerging, and drug-resistant infectious agents maximizes the importance of infection prevention and control. Healthcare workers are the key actors in the prevention and control of infection. As a result assessing the knowledge, attitude, and practice of healthcare workers toward infection prevention and control is very critical in the prevention and control of infectious diseases. Therefore, this systematic review and meta-analysis aimed to assess the knowledge, attitude, and practice of healthcare providers toward infection prevention in Ethiopia.

**Method:**

PubMed, Scopus, SEMANTIC SCHOLAR, Google Scholar, and Addis Ababa University Digital Library were systematically searched for relevant literature until November 18/2023. The quality of the included studies was assessed using the Joanna Briggs Institute quality appraisal tool. Data were abstracted using a Microsoft Excel spreadsheet and analyzed using STATA version 11. A random-effects model was used to estimate the pooled prevalence. Heterogeneity among reported studies was assessed by Forest plot, Cochran’s Q-statistics, and I^2^ test. Publication bias was checked using funnel plots, and Egger’s regression test. In addition, sub-group and sensitivity analyses were conducted.

**Result:**

A total of 7,681 articles were retrieved of which 19 studies with 5,650 healthcare workers were included in this systematic review and meta-analysis. About 74.5% (95% CI, 65.88, 83.12), 66.71% (95% CI 55.15, 78.28), and 55.2% (95% CI 48.22, 62.18) of healthcare workers were knowledgeable, had positive attitudes, and good standard of practice on infection prevention respectively.

**Conclusion:**

Despite acceptable knowledge and attitude, about half of the healthcare workers have unsafe infection prevention and control practices in Ethiopia. Hence, serious attention should be given to healthcare workers’ application of infection prevention standards in their working environment.

## Introduction

Infectious diseases are a global threat and the major cause of mortality and morbidity in human history [1, 2]. The emerging and re-emerging micro-organisms are continued as global challenges across generations [3, 4]. The new emerging micro-organisms like the COVID-19 virus are not only health problems, this has a multidimensional global catastrophe in human life that we are facing nowadays [5, 6]. The other challenges related to infectious diseases are the occurrence of drug-resistant pathogens, which become the disasters of humankind now and in the future [7, 8]. As a result, the eradication, elimination, prevention, and control of emerging and re-emerging infectious diseases are the global agendas [9–11]. Therefore, breaking the chain of transmission of infectious diseases is the main and cost-effective way even though it is challenging, especially in resource-limited countries [12–14].

For this reason providing effective, efficient, quality healthcare services for a community needs effective infection prevention and control practices [15]. Infection prevention and control practices are very cost-effective and simple approaches. These include the use of personal protective equipment, appropriate hand hygiene, environmental cleaning, instrument processing, safe injection, and proper infectious waste disposal management in healthcare facilities [16]. Infection prevention not only protects infectious diseases but also maximizes patients’ safety and protects health workers from different work-related hazards [17, 18].

For full implementation of infection prevention and control strategies, healthcare workers and health facilities are the main actors in the transmission and control of infectious diseases. The knowledge, attitude, and practices of healthcare workers are the determining factors for their well-being and that of their clients. In addition to that healthcare settings are one of the most common sources of infectious disease unless carefully established and well-managed [19]. The problems worsened in less developed countries with poor infrastructures, inadequate supply of infection prevention material, and ineffective prevention policies [20, 21]. For this reason, most preventable mortality and morbidity increased in alarming ways [22]. Infection prevention and control strategies are the most cost-effective approach to breaking the chain of infectious diseases, particularly for less developed countries like Ethiopia.

Studies conducted in low-income countries reported that the prevalence of healthcare-associated infections (HAIs) ranges from 5.7% to 19.1% [23]. The other study also showed that the incidence ranges from 5.7% to 45.8% [24]. Further evidence reported that general compliance with infection prevention recommendations among healthcare workers (HCWs) in many low-incoming countries is poor [25–27]. In Ethiopia, the burden of HAIs is a major public health problem with a significant impact on the mortality and morbidity of hospitalized patients (14–16). According to the findings of studies in Ethiopia, a high prevalence of HAIs has been observed from different parts of the country from 11.4%-19.4% in southwest Ethiopia (16, 17), 15.4% in north Ethiopia (15), to 16.4% in central Ethiopia (18). A systematic review and meta-analysis conducted in Ethiopia on the magnitude of HAIs reported 16.96% [28].

Besides, these healthcare-acquired infections may be related to poor setup, inadequate healthcare provider knowledge, unfavorable attitude, and malpractice [29]. Particularly, poor knowledge, unfavorable attitudes, and poor infection prevention and control practices among healthcare workers are risks for the healthcare provider and clients utilizing the service and for the environment [30]. Many people become infected in the healthcare setting, and especially, a large number of healthcare providers are exposed to infections from their clients [31, 32]. As evidenced by different literature, healthcare workers are the front-line to be exposed to a healthcare-related infection that leads to mortality and morbidity, and also they can be the source of infection for their clients [33, 34]. For this reason, the knowledge, attitude, and practice of healthcare workers are very important in the prevention and control of infection, especially in healthcare settings. Because healthcare workers are the backbone of the healthcare system, as a result, their knowledge, attitude, and practice toward infection prevention and control can majorly affect the public at large [35].

Despite this fact, the knowledge, attitude, and practice of healthcare workers toward infection prevention are not as needed in low incoming countries. In Ethiopia, different primary studies have been reported regarding the knowledge, attitude, and practice of healthcare workers toward infection prevention [36–54]. As the finding shows significant inconsistency among the studies, they are small-scale and single institutions, which may not represent the national figure of the topic. In addition to the above, there are no national surveys that assess the knowledge, attitude, and practice and their determinants among healthcare providers in Ethiopia. In general, there are evidence gaps in the level of knowledge, attitude, and practice of healthcare workers in Ethiopia. Hence, the findings of this systematic review and meta-analysis are expected to fill the evidence gaps for policymakers, healthcare workers, and other stakeholders to make evidence-based decisions and resource allocation to maximize patient and health worker safety. Furthermore, the findings will provide insight into the level of infection prevention awareness, readiness, and implementation status among healthcare workers in Ethiopia. As a result, assessing the knowledge, attitude, and practice of healthcare workers is expected to have a significant impact on infection prevention and control of emerging and re-emerging infectious diseases and drug-resistant microbes. Therefore, this systematic review and meta-analysis aimed to assess the pooled prevalence of healthcare workers’ knowledge, attitude, and practice toward infection prevention in Ethiopia.

## Method

### Protocol registration and reporting

The protocol of this systematic review and meta-analysis has been registered in the International Prospective Register of Systematic Review and Meta-analysis (PROSPERO) with a registration number CRD42022300672. This systematic review and meta-analysis are conducted following the Preferred Reporting Items for Systematic Reviews and Meta-Analysis (PRISMA) guidelines (S1 Checklist) [55].

### Eligibility criteria

The inclusion criteria for this study were the following: (1) Studies reported at least one of the following, knowledge, attitude, and practice toward infection prevention among healthcare workers in Ethiopia, (2) Study designs were observational, (3) studies had been performed on healthcare workers (4) both published and unpublished articles (like thesis) were included. The exclusion criteria of the study were the following: (1) studies conducted on populations other than healthcare workers (2) studies that were qualitative, reviews, case reports, and letters to editors.

### Information sources and search strategy

A comprehensive search and document retrieval strategy were performed to find potentially relevant published and unpublished articles in the following databases: PubMed, Scopus, and SEMANTIC SCHOLAR. In addition, the reference lists of all included articles were considered and searched. Google Scholar and manual search were conducted for gray literature. The full electronic search strategy was searched using the following search terms: knowledge, attitude, practice, “healthcare worker”, “healthcare personnel”, “healthcare professionals” “infection prevention” “infection control”, “standard precaution” and “Ethiopia”. A combination of appropriate Boolean operators (AND, OR), and truncation was used. The search for articles was conducted until November 18, 2023. EndNote X7 (Thomson Reuters, New York, USA) software was used to manage identified and retrieved studies (S1 File).

### Study selection

All retrieved articles from the search strategy were imported to EndNote X7. After removing the duplicated articles from EndNote Library, the titles and abstracts of the remaining articles were assessed independently by four reviewers (CAW, BDM, KAA, and MAT). The full-text articles were reviewed to confirm the eligibility criteria. Disagreements between the reviewers were resolved through discussion by involving other co-authors. The PRISMA flow diagram was used to summarize the study selection processes.

### Data collection process and main data items

All required data from included articles were extracted using a standardized, pre-piloted data extraction format. The four reviewers (CAW, BDM, KAA, and MAT) independently extracted the data using the Joanna Briggs Institute (JBI) data extraction form [56]. Disagreement during data extraction was resolved through discussion and consensus. Study characteristics such as first authors’ name, region, study year, publication year, study design, study setting, participants, sample size, data collection technique, sampling technique, response rate, and outcome measures (knowledge, attitude, and practice of healthcare workers on infection prevention) were extracted from the included studies.

### Quality assessment

The methodological quality and risk of bias of each included article were assessed by six authors (CAW, MAT, GMB, TGA, BDM, and KAA) independently using the JBI critical appraisal checklist [57]. Any disagreements during the quality assessment were resolved through discussion involving other co-authors. Studies that scored > 60% of the quality scores were considered good quality score and were included in the final systematic review and meta-analysis (S2 File).

### Measurement of outcome variables

The outcomes of this systematic review and meta-analysis were the knowledge, attitude, and practice of healthcare workers toward infection prevention. Infection prevention knowledge, attitude, and practices of healthcare workers were assessed for main components of infection prevention measures like utilization of personal protective equipment, hand hygiene practices, safe handling of waste material, injection safety, and disinfection practice. The three main outcome variables were considered as having good knowledge, favorable attitude, and good infection prevention practice as reported from each primary study.

### Data processing and analysis

The individual primary studies were described succinctly using a summary table. Healthcare workers’ knowledge, attitude, and practice toward infection control were computed using STATA version 11. A random-effects model was used to pool the estimate of knowledge, attitude, and practice of healthcare workers toward infection prevention. Findings were presented using forest plot graphical representation with a 95% confidence interval (CI), and Cochran’s Q test and I^2^ were used to detect heterogeneity between the studies. In addition, evidence of publication bias was checked using funnel plots asymmetry, and weighted Egger’s regression test with a p-value of less than 0.05 as a cutoff point to declare the presence of publication bias. Considering the observed heterogeneity between included studies sub-group and sensitivity analysis were performed to identify potential source of variations.

## Result

### Study selection

**Fig 1:**
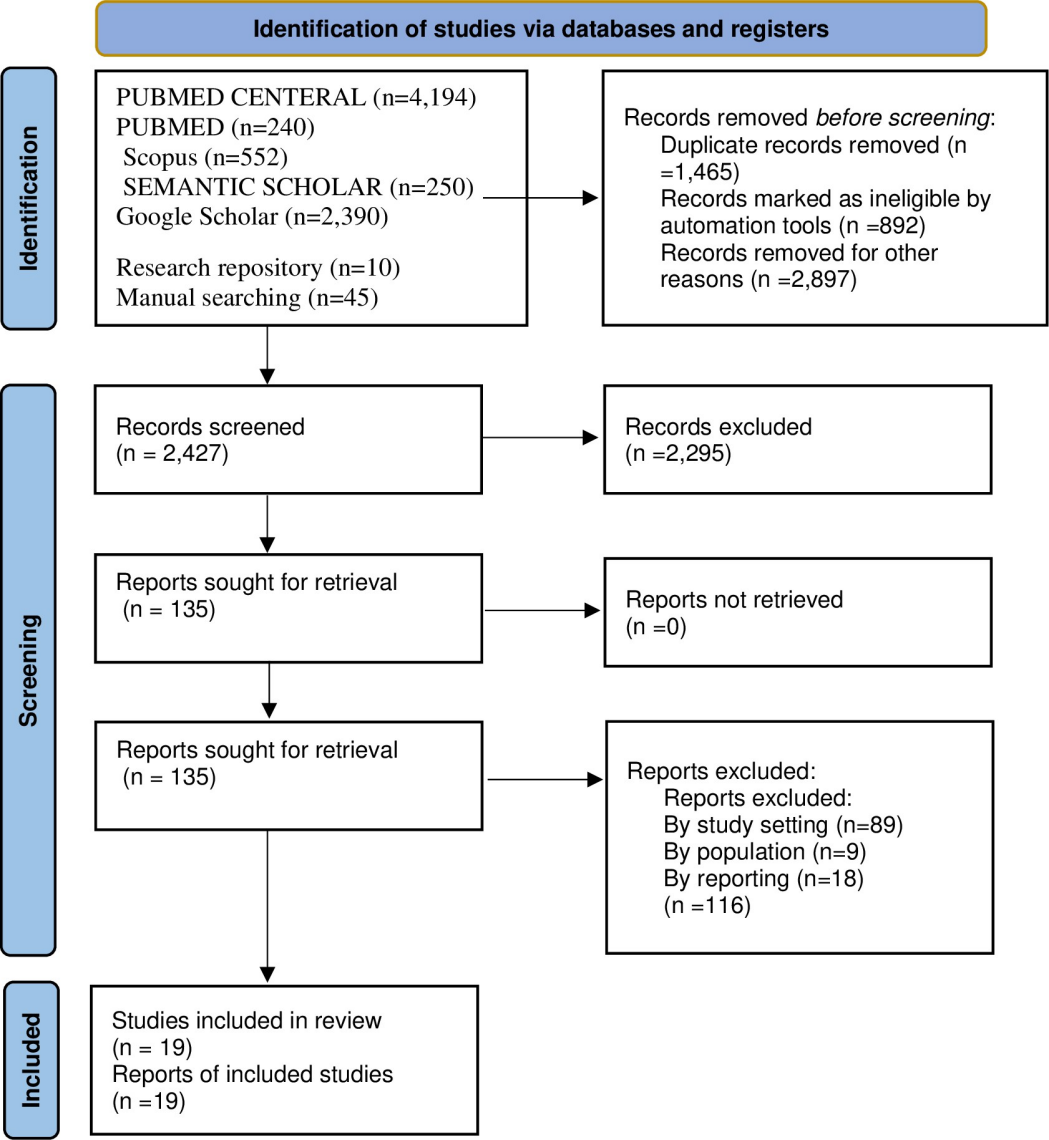
Flow diagram of articles selection and screening process.

Through comprehensive searching, 7,681 articles were identified. Three thousand six hundred sixty-seven studies were excluded due to duplications. One thousand thirty-five studies were assessed for the title, abstracts, and full paper. Finally, 19 papers consisting of 5,650 were included in this systematic review and meta-analysis. All 19 included studies were used to pool the practice of infection prevention, 17 for knowledge, and 9 articles for attitude of healthcare workers towards infection prevention (Fig 1).

### Characteristics of included studies

All included studies were cross-sectional studies and published between 2014 and 2021. Regarding the study setting nine studies were conducted in the Amhara region [37, 39, 40, 42, 44, 45, 51–53] followed by five studies in the Oromia region [41, 43, 47, 49, 54] and the remaining two were from Addis Ababa, one from Afar, Tigray, and SNNPR. The majority of included studies were conducted among all healthcare workers, whereas three studies were conducted by involved only nurses [36, 38, 41]. Except for two studies which is a thesis (35, 51), all included studies are published studies in peer-reviewed journals. All the included studies vary in their sample size ranges from 91–680 [43, 48]. Regarding the data collection technique majority (13 articles) used self-administered questioners [36, 38, 40–49, 52, 53], 2 self-administered questioners with observation [45, 51], three articles used interview administered [39, 50, 54] and the remaining one article did not mention the technique [37]. Nearly half of the included studies were conducted using a simple random sampling technique [37, 40, 41, 44, 45, 54], three systematic random samplings [40, 47, 52], two stratified samplings [49, 51], and two multistage [43, 50] (Table 1).

**Table 1:** Descriptive summary of primary studies included in the systematic review and meta-analysis of knowledge, attitude, and practice toward infection prevention among healthcare workers in Ethiopia.

First author	Year of publication	Region	Study area	Study design	Study population	Sample size	Response rate (%)	Knowledge (%)	Attitude (%)	Practice (%)	Data collection technique
Sahiledengle, B., et al.	2018	Addis Ababa	Governmental healthcare facilities	Cross sectional	All health workers	629	96.2	55.4	83.3	66.115	Interview
Yazie et al.	2019	Amhara	Gondar University referral hospital	Cross-sectional	Healthcare provider	282	100	81.56	64.18	57.45	Self-administer questioner
Zenbaba et al.	2020	Oromia	Bale zone hospitals	Cross-sectional	All health workers	402	98	72.08	-	36.8	Interview
Alemayehu et al.	2016	Amhara	Dessie Referral Hospital	Cross-sectional	All health workers	208	100	95.19	-	87.5	Not mentioned
Gezie H, et al.	2019	Amhara	Dessie Referral Hospital	Cross-sectional	All health workers	211	90.5	86.38	76.44	23.03	Self-administer questioner
Geberemariyam et al.	2018	Oromia	Arsi District	Cross-sectional	All health workers	680	95.3	53.7	-	36.26	Self-administer questioner
Desta et al.	2018	Amhara	Debre Markos referral hospital	Cross-sectional	All health workers	158	95	84.67	-	57.33	Self-administer questioner
Gulilat k et al	2014	Amhara	Bahir Dar city Health institution	Cross sectional	All health workers	362	97.8	84.46	55.65	54.24	Self-administer questioner &observation
Hussein, S., et al.	2017	SNNPR	Wolaitta Sodo Otona Teaching and Referral Hospital	Cross-sectional	All health workers	282	95.7	99.26	93.36	60.52	Self-administer questioner
Bekele et al.	2018	Oromia	Jimma University Medical Center	Cross-sectional	Nurses	231	100	93.07	-	64.07	Self-administer questioner
Yallew WW et al.	2017	Amhara	University of Gondar Hospital and Felege-Hiwot Hospital	Cross-sectional	All health workers	422	97.9	-	-	54.96	Self-administer questioner
Assefa et al.	2020	Amhara	Wogdie District/South Wollo	Cross-sectional	All health workers	171	100	70.76	-	54.97	Interview
Jemal, K., et al.	2020	Oromia	North Showa Health facility	Cross-sectional	All health workers	373	96.8	58.2	59.28	46.81	Self-administer questioner
Melesse GT, et al.	2018	Oromia	West Guji Zone Health facility	Cross-sectional	All health workers	203	99	59.7	40.8	54.73	Self-administer questioner
Bayleyegn et al	2021	Amhara	Gondar University referral hospital	Cross-sectional	All health workers	236	100	90.25	57.2	63.98	Self-administer questioner
Jemal, S., et al.	2019	Afar	Dubti referral hospital	Cross-sectional	All health workers	91	100	50.55	69.23	48.35	Self-administer questioner
Worede, D.T., et al.	2021	Amhara	South Gondar zone Health fasilities	Cross-sectional	All health workers	620	97	-	-	60.79	Self-administer questioner
Abreha, N.	2018	Addis Abeba	Tikur Anbessa Specialized Teaching Hospital	Cross-sectional	Nurse	108	94.4	75.49	-	72.55	Self-administer questioner
Asfaw. N	2021	Tigray	Aksum Saint Mary hospital	Cross-sectional	Nurse	143	97.2	52.52	-	48.2	Self-administer questioner

### Knowledge of healthcare workers on infection prevention and control

**Fig 2:**
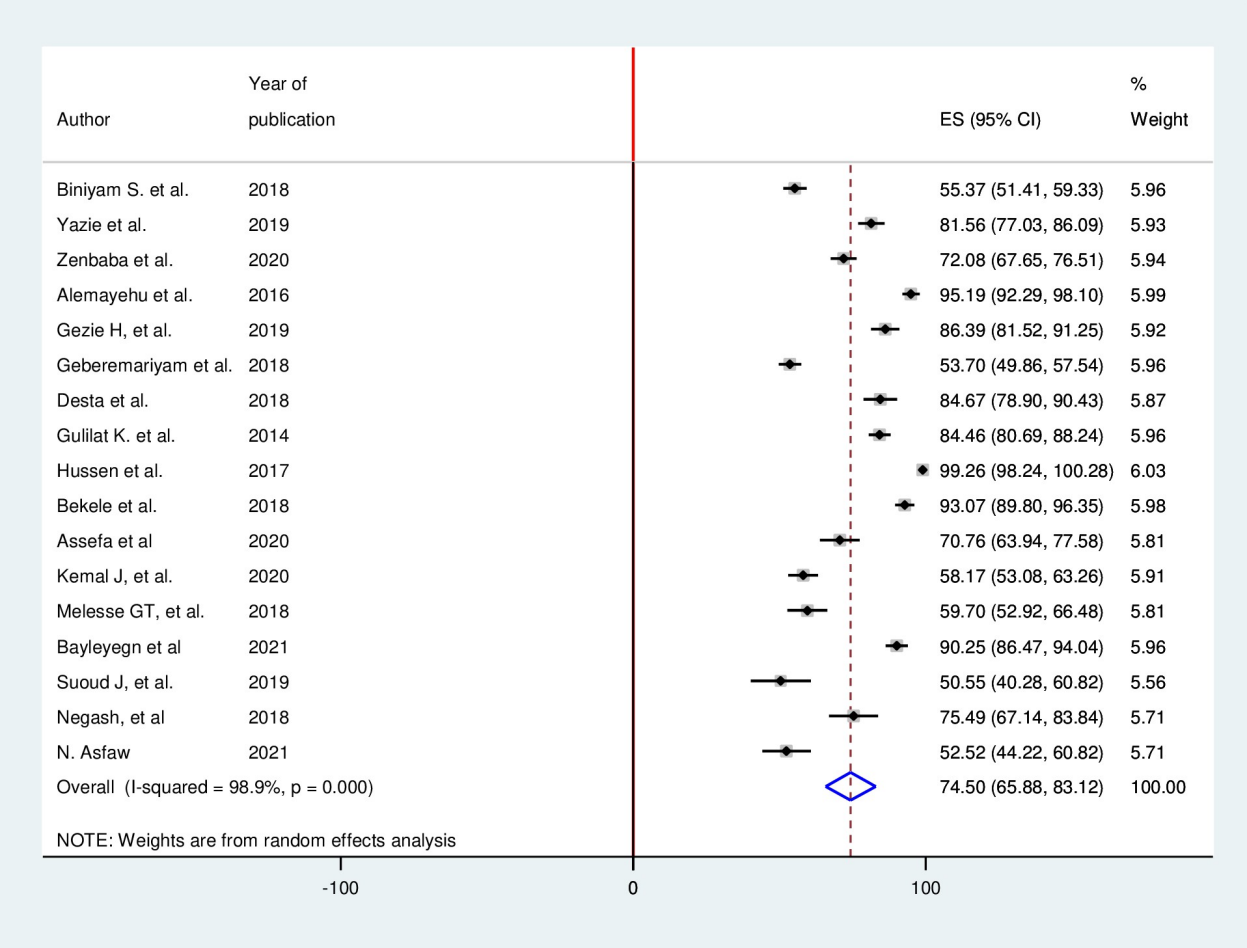
Forest plot for the proportion of knowledge of health care works regarding infection prevention.

In this systematic review and meta-analysis, 17 studies have reported knowledge of healthcare works toward infection prevention with 4,770 study participants [36, 37, 39–50, 53, 54]. This systematic review and meta-analysis reported that 74.5% (95% CI 65.88, 83.12) of healthcare professionals were knowledgeable regarding infection prevention in Ethiopia. Heterogeneity among included studies was observed (I^2^ = 98.9%; p<0.001). For this reason, random-effect, model was used to estimate the pooled prevalence of knowledge of healthcare workers (Fig 2).

### Attitude of healthcare workers toward infection prevention and control

**Fig 3:**
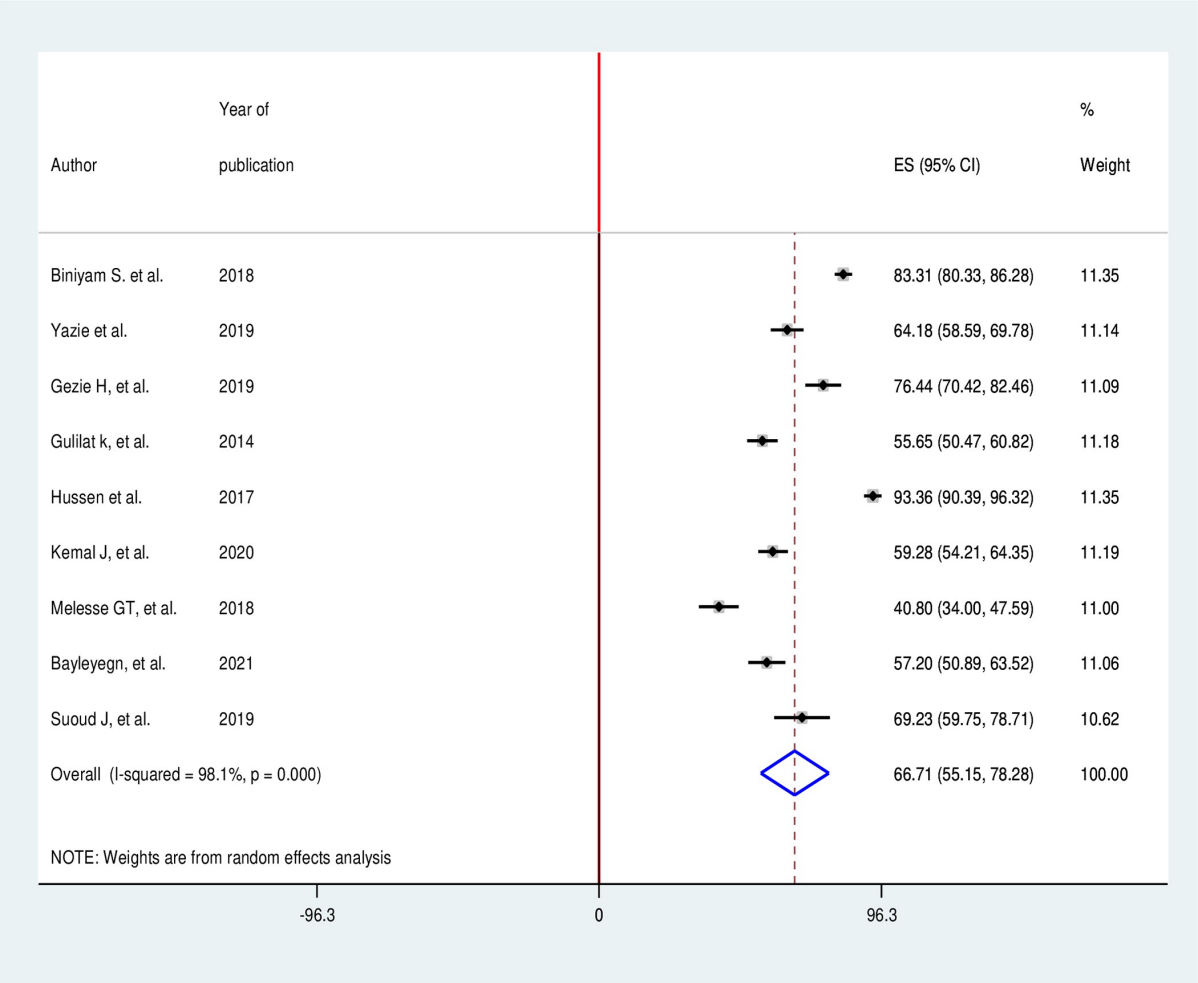
Forest plot for the proportion of health care workers attitude towards infections preventions.

After reviewing different articles 9 research papers fulfilled the inclusion criteria for final analysis in the attitude part for meta-analysis and systematic review [40, 44–50, 53]. The finding revealed that 66.71% (95% CI 55.15, 78.28) of healthcare workers had a favorable attitude towards infection prevention to prevent healthcare-associated infections. From these nine analyzed studies, 2,387 healthcare workers participated. Heterogeneity between included studies was assessed and it was statistically significant (I^2^ = 98.1%; p<0.001). By observing this, a random-effect model was applied to estimate the prevalence (Fig 3).

### Healthcare workers practice toward infectious prevention and control

**Fig 4:**
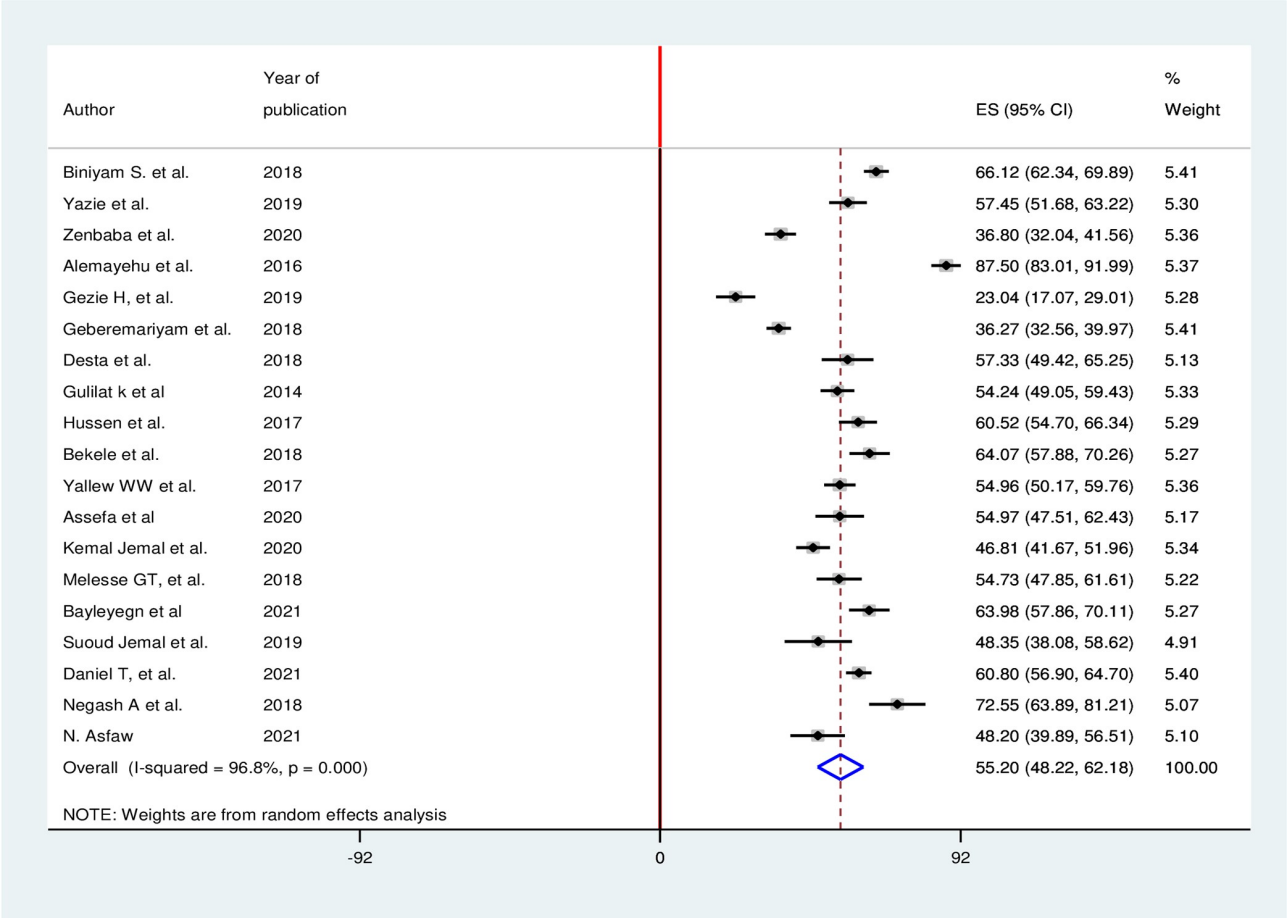
Forest plot for the proportion of practice of health care works regarding infection prevention.

A total of 19 studies have been included in the systematic review and meta-analysis [36–54]. Compared with infection prevention knowledge and attitude, a large proportion of articles reported regarding infectious prevention practices among healthcare workers. A total of 5,650 study participants were involved in this systematic review and meta-analysis from 19 studies included in the practice. From these study participants, more than half, 55.2% (95% CI 48.22, 62.18) of healthcare workers had good practices towards infection prevention in the healthcare facilities in Ethiopia. Random-effect model was used to estimate the prevalence of infection prevention practice since significant heterogeneity among included studies was observed (I^2^ = 98.1%; p<0.001) (Fig 4).

### Publication bias

Publication bias was assessed using a funnel plot and Egger’s test for the three outcomes (knowledge, attitude, and practice). The result showed no evidence of publication bias was observed for practice (p-values of 0.966). However, there is significant evidence of publication bias for knowledge (p-value of <0.001) and attitude (p-value of 0.012) (S3 File).

### Subgroup analysis

Sub-group analysis was conducted using different variables for knowledge, attitude, and practice. The sub-group analysis was conducted by considering region, profession, sample size, and year of publication. The result showed that the highest prevalence of good knowledge was observed in Amhara region 85.12 (95%CI: 79.86, 90.38), among all healthcare workers 74.60 (95%CI: 64.73, 84.46), sample size less than 350 78.94 (95%CI: 71.67, 86.21) and studies published before 2020, 76.83 (95%CI: 66.64, 87.01) among healthcare workers regarding infection prevention. Regarding the attitude of healthcare workers towards infection prevention, the highest prevalence of good attitude was reported from other regions (Addis Ababa, Afar, and SNNP) 83.00(95%CI: 72.98, 93.02) and studies conducted and published before 2020, 69.14(95%CI: 55.97, 82.31). And better infection prevention practice was reported in Addis Ababa 68.10(95%CI: 62.28, 73.93), professionally among nurses only 61.62(95%CI: 48.81, 74.42), sample size less than 350 and year of publication before 2020, 56.71(95%CI: 47.20, 66.21) among healthcare workers. Detailed description of the sub-group analysis can be observed in Table 2.

**Table 2:** Sub-group analysis of knowledge, attitude, and practice toward infection prevention among healthcare workers in Ethiopia.

**1. Knowledge**						
Variables	Subgroup	Number of included studies	Sample size	Prevalence (95% CI)	Heterogeneity across the studies	
					I^2^ (%)	P-value
Study conducted region	Amhara	7	1,569	85.12(79.86, 90.38)	90.4	p<0.001
	Oromia	5	1,835	67.41(50.72, 84.10)	98.6	p<0.001
	Others(Afar, Tigray and SNNP)	3	501	67.66(29.67, 105.65)	99.0	p<0.001
	Addis Ababa	2	707	68.08(45.38, 84.79)	94.5	p<0.001
Profession	All health workers	14	4,163	74.60(64.73, 84.46)	99.1	p<0.001
	Nurse only	3	472	73.90(49.21, 98.59)	97.7	p<0.001
Sample size	<350	12	2,273	78.94(71.67, 86.21)	97.7	p<0.001
	≥350	5	2,362	64.77(52.46, 7709)	97.7	p<0.001
Year of publication	<2020	12	3,331	76.83(66.64, 87.01)	99.0	p<0.001
	≥ 2020	5	1,301	68.95(55.29, 82.62)	97.0	p<0.001
**2. Attitude**						
Study conducted region	Amhara	4	962	63.33(54.19, 72.48)	90.01	p<0.001
	Others(Addis Ababa, Afar and SNNP)	3	949	83.00(72.98, 93.02)	94.6	p<0.001
	Oromia	2	562	50.18(32.07, 68.29)	94.5	p<0.001
Sample size	<350	6	1,272	66.95(49.74, 84.16)	98.3	p<0.001
	≥350	3	1,320	66.17(46.98, 85.36)	98.3	p<0.001
Year of publication	<2020	7	1,764	69.14(55.97, 82.31)	98.2	p<0.001
	≥ 2020	2	597	58.47(54.51, 62.42)	0	0.615
**3. Practice**						
Study conducted region	Amhara	9	2,607	57.19(46.04,68.35	97.4	p<0.001
	Oromia	5	1,835	47.53(37.51,57.54)	94.8	p<0.001
	Others(Afar, Tigray and SNNP)	3	501	52.97(43.95,61.99)	73.8	0.022
	Addis Ababa	2	707	68.10(62.28,73.93)	43.9	0.182
Profession	All healthcare workers	16	5,178	54.02(46.24,61.81)	97.2	p<0.001
	Nurse only	3	472	61.62(48.81, 74.42)	88.0	p<0.001
Sample size	<350	12	2,273	57.78(47.51, 68.06)	96.6	p<0.001
	≥350	7	3,374	50.87(41.63, 60,.11)	96.8	p<0.001
Year of publication	<2020	13	3,747	56.71(47.20, 66.21)	97.4	p<0.001
	≥ 2020	6	1,903	51.92(42.90, 60.94)	93.6	p<0.001

### Sensitivity analysis

Sensitivity analysis has been conducted to identify the possible source of the heterogeneity. The results of the sensitivity analysis showed that the findings are not dependent on a single study for the three outcomes. The pooled estimated prevalence of knowledge, attitude and infection prevention practice varied between 74.5% (95% CI: 65.88, 83.12) and 75.83 (95% CI: 67.12, 84.54), 66.71% (95% CI 55.15, 78.28) and 66.40 (95% CI: 53.96, 78.85), and 55.2 (95% CI: 48.22, 62.18) and 55.57 (95% CI: 48.33, 62.81) respectively after removing a single study (Table 3).

**Table 3:** Sensitivity analysis of knowledge, attitude, and practice toward infection prevention among healthcare workers in Ethiopia.

Study omitted	Prevalence of practice (95% CI)	Prevalence of knowledge (95% CI)	Prevalence of attitude (95% CI)
Sahiledengle, B., et al.(2018)	54.57 (47.22, 61.92)	75.74 (7.52, 83.97)	64.57 (50.75, 78.39)
Yazie et al.(2019)	55.07 (47.71, 62.42)	74.04 (64.95, 83.14)	67.022 (54.33, 79.70)
Zenbaba et al. (2020)	56.23 (49.21, 63.26)	74.65 (65.70, 83.59)	-
Alemayehu et al.(2016)	53.34 (47.45, 59.23)	73.16 (63.69, 82.62)	-
Gezie H, et al.(2019)	56.99 (50.45, 63.53)	73.74 (64.65, 82.83)	65.48 (52.63, 78.33)
Geberemariyam et al.(20018)	56.28 (49.48, 63.07)	75.86 (67.80, 83.92)	-
Desta et al.(2018)	55.08 (47.81, 62.34)	73.86 (64.83, 82.88)	-
Gulilat k et al(2014)	55.25 (47.84, 62.65)	73.85 (64.63, 83.07)	68.11 (56.04, 80.17)
Hussein, S., et al.(2017)	54.89 (47.56, 62.23)	72.94 (64.89, 81.00)	63.31 (52.76, 73.87)
Bekele et al.(2018)	54.70 (47.42, 61.98)	73.30 (63.94, 82.66)	-
Yallew WW et al.(2017)	55.20 (47.76, 62.64)	-	-
Assefa et al.(2020)	55.20 (47.92, 62.49)	74.73 (65.83, 83.62)	-
Jemal, K., et al.(2020)	55.66 (48.33, 63.00)	75.53 (66.93, 84.14)	67.64 (55.23, 80.05)
Melesse GT, et al.(2018)	55.22 (47.92, 62.52)	75.41 (66.66, 84.17)	69.93 (58.93, 80.94)
Bayleyegn et al.(2021)	54.70 (47.42, 61.99)	73.48 (64.24, 82.73)	67.89 (55.63, 80.16)
Jemal, S., et al. (2019)	55.55 (48.34, 62.75)	75.91 (67.16, 84.65)	66.40 (53.96, 78.85)
Worede, D.T., et al.(2021)	54.87 (47.36, 62.39)	-	-
Abreha, N. (2018)	54.27 (47.12, 61.41)	74.43 (65.52, 83.35)	-
Asfaw. N(2021)	55.57 (48.33, 62.81)	75.83 (67.12, 84.54)	-

## Discussion

Infection prevention is the most cost-effective approach to the reduction of morbidity and mortality in healthcare settings. Healthcare-associated infections are the most severe and worst form of infection than community-acquired. Healthcare workers are the most vulnerable group as well as a potential source of infection for their clients. Therefore, the knowledge, attitude, and practice of healthcare workers are crucial in the prevention of healthcare-associated infections. This issue is very mandatory action in the era of emerging and re-emerging infectious diseases like COVID-19 and drug-resistant microbial.

This systematic review and meta-analysis revealed that the knowledge of healthcare workers about infection prevention is 74.5% (95% CI, 65.88, and 83.12). The other outcome of this study is the attitude of healthcare workers regarding infection prevention. The magnitude of healthcare workers attitude is 66.71% (95% CI, 55.15, and 78.28) towards infectious prevention.

The third outcome of this systematic review and meta-analysis is infection prevention practice among healthcare workers in Ethiopia’s healthcare facilities. The finding revealed that 55.20% (95% CI, 48.22, and 62.18) of healthcare workers practice infection prevention activity in the healthcare facility in the recommended way. The finding of this study is in line with previously conducted systematic review and meta-analysis in Ethiopia which 52.2% of the healthcare workers had good practice towards infection prevention and control [58].

This systematic review and meta-analysis revealed that the majority of healthcare workers had adequate knowledge and a better attitude toward infection prevention in health facilities. However, about half of the healthcare workers are properly practicing infection prevention practice. This implies that having satisfactory knowledge and attitude alone is not guaranteed to implement infection prevention practices. Acceptable level of knowledge and attitude toward infection prevention may be the precondition for evidence-based infection prevention practice. The reason for poor implementation of infection prevention despite better knowledge and attitude may be external factors related to the institutions or beyond the institutions. The facility-related factors may be poor infrastructures like supply of water, sanitizer, and lack of personal protective equipment, lack of clear institutional and national infection prevention policy, poor regulatory health system, and poor motivation. Beyond this, there may be also problems in the knowledge and attitude of administrative bodies towards infection prevention practice that can be a challenge for the implementation of safe infection prevention practice [59–61].

On the other way, there may be personal commitment problems despite having good knowledge regarding infection prevention [62]. The finding shows a decline in the proportion of knowledge, attitude, and practice among healthcare workers about infection prevention in healthcare institutions in Ethiopia.

The presence of unsafe practices towards infection prevention is evidenced by a higher burden of healthcare-associated infection in Ethiopia. A systematic review and meta-analysis conducted in Ethiopia reported that 16.96% magnitude of healthcare-associated infection [28, 63].

The limitation of this systematic review and meta-analysis is the high variability among measurement tools across primary studies. This may affect the outcome of the study and could be one source of bias. The other limitation of this study is due to inconsistent reporting on associated factors, we are unable to determine factors associated with knowledge, attitude, and practice.

## Conclusion

This systematic review and meta-analysis reported that the majority of healthcare workers have adequate knowledge and a better attitude toward infection prevention. Despite this satisfactory knowledge and attitude, about half of the healthcare workers have unsafe infection prevention and control practices. Possible challenges and reasons for unsafe implementation of infection prevention and control practices among healthcare workers shall be investigated. Efforts to improve infection prevention and control practices among healthcare workers shall be a priority health agenda in Ethiopia.

## Supporting information

S1 Checklist Preferred Reporting Items for Systematic Review and Meta-analysis (PRISMA) guideline. (DOCX)

S1 File Search strategy and terms in PubMed. (DOCX)

S2 File JBI critical appraisal checklist. (DOCX)

S3 File Funnel plot for publication biases. (DOCX)
