# Supplementary material for: Knowledge, attitude and practice of healthcare workers on infection prevention and control in Ethiopia: A systematic review and meta-analysis
Source: PLoS One. 2024 Sep 5;19(9):e0308348. doi: 10.1371/journal.pone.0308348 (PMC11376544; doi:10.1371/journal.pone.0308348)
Supplement: S1 Checklist — (DOCX) [file pone.0308348.s001.docx]

**Supplementary File 1:** Preferred Reporting Items for Systematic Review and Meta-analysis (PRISMA) guideline.

**Checklist**
