# Supplementary material for: Knowledge, attitude and practice of healthcare workers on infection prevention and control in Ethiopia: A systematic review and meta-analysis
Source: PLoS One. 2024 Sep 5;19(9):e0308348. doi: 10.1371/journal.pone.0308348 (PMC11376544; doi:10.1371/journal.pone.0308348)
Supplement: S1 File — (DOCX) [file pone.0308348.s002.docx]

**Additional file 2 Search strategy and terms in PubMed**

|  | **Area** | **Query** |
| --- | --- | --- |
| **#1** | **Infection prevention knowledge, attitude and practice** | “Knowledge”, “awareness”, “attitude”, “perception”, “practice”, “preventive measure*” |
| **#2** | **Population** | “healthcare worker*”, “healthcare personnel*”, “healthcare professional*”, “healthcare provider*” |
| **#3** | **Country** | Ethiopia |
| **#4** | **Limits** | Up to November 18/2023 |

**Example of PubMed search**

“Knowledge”, “awareness”, “attitude”, “perception”, “practice”, “preventive measure*”, “healthcare worker*”, “healthcare personnel*”, “healthcare professional*”, “healthcare provider*” “infection prevention”, “infection control”, “standard precaution”, “universal precaution”, “Ethiopia”.

**((((((((((((((Knowledge) OR (awareness)) AND (attitude)) OR (perception)) AND (practice)) OR ("preventive measure*")) AND ("healthcare worker*")) OR ("healthcare personnel*")) OR ("healthcare professional*")) OR ("healthcare provider*")) AND ("infection prevention")) OR ("infection control")) OR ("standard precaution")) OR ("universal precaution")) AND (Ethiopia)**

((((((((("knowledge"[MeSH Terms] OR "knowledge"[All Fields] OR "knowledge s"[All Fields] OR "knowledgeability"[All Fields] OR "knowledgeable"[All Fields] OR "knowledgeably"[All Fields] OR "knowledges"[All Fields] OR ("awareness"[MeSH Terms] OR "awareness"[All Fields] OR "aware"[All Fields] OR "awarenesses"[All Fields])) AND ("attitude"[MeSH Terms] OR "attitude"[All Fields] OR "attitudes"[All Fields] OR "attitude s"[All Fields])) OR ("percept"[All Fields] OR "perceptibility"[All Fields] OR "perceptible"[All Fields] OR "perception"[MeSH Terms] OR "perception"[All Fields] OR "perceptions"[All Fields] OR "perceptional"[All Fields] OR "perceptive"[All Fields] OR "perceptiveness"[All Fields] OR "percepts"[All Fields])) AND ("practicability"[All Fields] OR "practicable"[All Fields] OR "practical"[All Fields] OR "practicalities"[All Fields] OR "practicality"[All Fields] OR "practically"[All Fields] OR "practicals"[All Fields] OR "practice"[All Fields] OR "practice s"[All Fields] OR "practiced"[All Fields] OR "practices"[All Fields] OR "practicing"[All Fields])) OR "preventive measure*"[All Fields]) AND "healthcare worker*"[All Fields]) OR "healthcare personnel*"[All Fields] OR "healthcare professional*"[All Fields] OR "healthcare provider*"[All Fields]) AND "infection prevention"[All Fields]) OR "infection control"[All Fields] OR "standard precaution"[All Fields] OR "universal precaution"[All Fields]) AND ("ethiopia"[MeSH Terms] OR "ethiopia"[All Fields] OR "ethiopia s"[All Fields])
