# Supplementary material for: Knowledge, attitude and practice of healthcare workers on infection prevention and control in Ethiopia: A systematic review and meta-analysis
Source: PLoS One. 2024 Sep 5;19(9):e0308348. doi: 10.1371/journal.pone.0308348 (PMC11376544; doi:10.1371/journal.pone.0308348)
Supplement: S2 File — (DOCX) [file pone.0308348.s003.docx]

**Supplementary File 3:** JBI critical appraisal checklist

| Articles | Critical appraisal criteria for cross-sectional studies | | | | | | | | | | | | | | | | | | | | | | | | Total score |
| --- | --- | --- | --- | --- | --- | --- | --- | --- | --- | --- | --- | --- | --- | --- | --- | --- | --- | --- | --- | --- | --- | --- | --- | --- | --- |
|  | Were the criteria for inclusion in the sample clearly defined? | | | Were the study subjects and the setting described in detail? | | | Was the exposure measured in valid and reliable way? | | | Were objective, standard criteria used for measurement of the condition | | | Were confounding factors identified? | | | Were strategies to deal with confounding factors stated? | | | Were the outcomes measured in a valid and reliable way? | | | Was appropriate statistical analysis used? | | |  |
| First author | yes | No | NA | Yes | No | NA | Yes | No | NA | Yes | No | NA | Yes | No | NA | Yes | No | NA | Yes | No | NA | Yes | No | NA |  |
| Biniyam S. et al. | √ |  |  | √ |  |  | √ |  |  | √ |  |  |  | x |  |  | × |  | √ |  |  | √ |  |  | 75% |
| Yazie et al. | √ |  |  | √ |  |  | √ |  |  |  | x |  |  | x |  |  | × |  | √ |  |  | √ |  |  | 62.5% |
| Zenbaba et al. | √ |  |  | √ |  |  |  | × |  | √ |  |  | √ |  |  |  | × |  | √ |  |  | √ |  |  | 75% |
| Alemayehu et al. |  | x |  | √ |  |  | √ |  |  | √ |  |  |  | x |  |  | × |  | √ |  |  | √ |  |  | 62.5% |
| Gezie H, et al. | √ |  |  | √ |  |  | √ |  |  | √ |  |  | √ |  |  |  | × |  | √ |  |  |  | x |  | 75% |
| Geberemariyam et al. | √ |  |  | √ |  |  | √ |  |  | √ |  |  |  | x |  |  | × |  | √ |  |  | √ |  |  | 75% |
| Desta et al. | √ |  |  | √ |  |  | √ |  |  | √ |  |  |  | × |  |  | × |  | √ |  |  | √ |  |  | 75% |
| Gulilat k et al | √ | x |  | √ |  |  |  | × |  | √ |  |  |  | × |  |  | × |  | √ |  |  | √ |  |  | 62.2% |
| Hussen et al. |  | x |  | √ |  |  | √ |  |  | √ |  |  |  |  | × |  |  | × | √ |  |  | √ |  |  | 62.5% |
| Bekele et al. | √ |  |  | √ |  |  | √ |  |  | √ |  |  |  |  | × |  |  | × | √ |  |  | √ |  |  | 75% |
| Yallew WW et al. |  | x |  | √ |  |  | √ |  |  | √ |  |  | √ |  |  | √ |  |  | √ |  |  | √ |  |  | 87.5% |
| Assefa et al. | √ |  |  | √ |  |  |  | x |  | √ |  |  |  | x |  | √ |  |  | √ |  |  | √ |  |  | 75% |
| Kemal Jemal et al. | √ |  |  | √ |  |  | √ |  |  | √ |  |  |  | x |  | √ |  |  | √ |  |  | √ |  |  | 87.5% |
| Melesse GT, et al. | √ |  |  | √ |  |  |  | x |  | √ |  |  |  |  | x |  |  | x | √ |  |  | √ |  |  | 62.5% |
| Bayleyegn et al | √ |  |  | √ |  |  |  | x |  | √ |  |  |  | x |  |  | x |  | √ |  |  | √ |  |  | 62.5% |
| Suoud Jemal et al. | √ |  |  | √ |  |  |  | x |  | √ |  |  |  |  | x |  |  | x | √ |  |  | √ |  |  | 62.5% |
| Daniel T, et al. | x |  |  | √ |  |  |  | x |  | √ |  |  |  | x |  | √ |  |  | √ |  |  | √ |  |  | 62.5% |
| Negash, et al. | √ |  |  | √ |  |  | √ |  |  | √ |  |  |  | x |  |  |  | x | √ |  |  | √ |  |  | 75% |
| Asfaw. N | x |  |  | √ |  |  | √ |  |  | √ |  |  |  | x |  | √ |  |  | √ |  |  | √ |  |  | 75% |
