# Supplementary material for: Knowledge, attitude and practice of healthcare workers on infection prevention and control in Ethiopia: A systematic review and meta-analysis
Source: PLoS One. 2024 Sep 5;19(9):e0308348. doi: 10.1371/journal.pone.0308348 (PMC11376544; doi:10.1371/journal.pone.0308348)
Supplement: S3 File — (DOCX) [file pone.0308348.s004.docx]

Funnel plot for practice

Funnel plot for attitude

Funnel plot for knowledge
